# A comparative study of two various models of organising diabetes follow-up in public primary health care – the model influences the use of services, their quality and costs

**DOI:** 10.1186/1472-6963-14-26

**Published:** 2014-01-20

**Authors:** Mikko T Honkasalo, Miika Linna, Timo Sane, Atte Honkasalo, Outi Elonheimo

**Affiliations:** 1 Nurmijärvi Health Centre, Network of Academic Health Centres, University of Helsinki, University Central Hospital of Helsinki, Unit of General Practice, Helsinki, Finland; 2 Health Care Management and Architecture, Aalto University, Helsinki, Finland; 3 Department of Endocrinology, University Central Hospital of Helsinki, Helsinki, Finland; 4 Qvantel Oy, Espoo, Finland; 5 Network of Academic Health Centres, University of Helsinki, University Central Hospital of Helsinki, Unit of General Practice, Helsinki, Finland

**Keywords:** Type 1 diabetes, Organisation of diabetes care, Costs of diabetes care, Patient satisfaction, Comparison of diabetes care, PHC diabetes care

## Abstract

**Background:**

In Finland diabetologists have long been concerned about the level of diabetes care as the incidence of type 1 diabetes and complicated type 2 diabetes is exceeding the capacity of specialist clinics. We compared the outcome of diabetes care in two middle-sized Finnish municipalities with different models of diabetes care organisation in public primary health care. In Kouvola the primary health care of all diabetic patients is based on general practitioners, whereas in Nurmijärvi the follow-up of type 1 and most complicated type 2 diabetic patients is assigned to a general practitioner specialised in diabetes care.

**Methods:**

Our study population consisted of all adult diabetic patients living in the municipalities under review.

We compared the use and costs of public diabetes care, glycemic control, blood pressure, LDL-cholesterol level, the application of the national guidelines and patient satisfaction. The main outcome measures were the costs and use of health care services due to diabetes and its complications.

**Results:**

In Nurmijärvi, where diabetes care was centralised, more type 1 diabetic patients were followed up in primary health care than in Kouvola, where general practitioners need more specialist consultations. The centralisation resulted in cost savings in the diabetes care of type 1 diabetic patients. Although the quality of care was similar, type 1 diabetic patients were more satisfied with their follow-up in the centralised system. In the care of type 2 diabetic patients the centralised system required fewer specialist consultations, but the quality and costs were similar in both models.

**Conclusions:**

The follow-up of most diabetic patients – including type 1 diabetes – can be organised in primary health care with the same quality as in secondary care units. The centralised primary care of type 1 diabetes is less costly and requires fewer specialist consultations.

## Background

Patients with common diseases like arterial hypertension, bronchial asthma or type 2 diabetes (T2D) are usually followed up by general practitioners (GP) in primary health care (PHC). However, there are some chronic diseases - such as type 1 diabetes (T1D) - with challenging treatment demands and increasing incidence. Finland has one of the highest incidences of T1D in the world [1]. In many municipalities, general practitioners in PHC have to take responsibility of all diabetic patients living in their district. The knowledge of diabetes care and the clinical experience of general practitioners may not meet the needs of their T1D patients and it is difficult to get enough clinical experience if the number of T1D patients under their follow-up care is very limited. According to recent data, the quality standard in the care of T1D patients remains unsatisfactory and there has not been any improvement in glycemic control during the past decades [2,3].

In Finland the municipalities, which pay the costs of the public health care of their inhabitants, are interested to produce high-quality care of common chronic diseases as cost-effectively as possible [4].

The quality of diabetes care between a diabetes clinic and a general medicine clinic has been compared [5], but not between PHC clinics with different models of care organisation. The reason for this may be the difficulty of planning an adequate study design.

**Figure 1:**
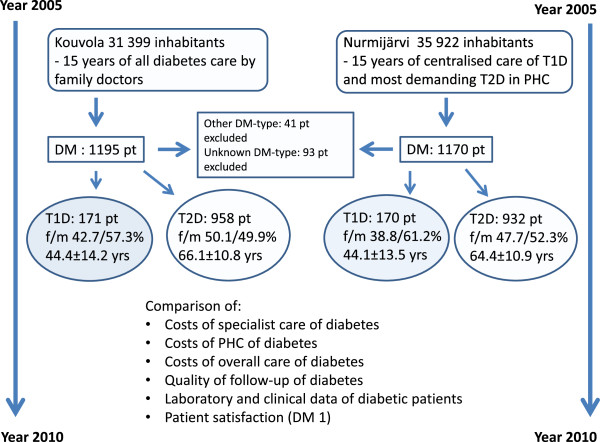
A flow chart of the course of the study.

In our study, we compared the outcomes and costs of diabetes care in two middle-sized Finnish municipalities, which have had different models in the care of diabetic patients in PHC for about 15 years –one is centralised, the other based on general practitioners (Figure 1). Our principal interest was the balance between the quality and costs of T1D patient care in these two municipalities. We studied the use of diabetes services both in PHC and in secondary HC, and their prices over a period of six years (2005–2010). We measured the clinical quality and coverage of diabetes care with clinical and biometric markers and the validity of the care with the proportion of patients followed up according to the Finnish standards of good medical practice in diabetes care [6]. Moreover, we asked the T1D patients how satisfied they were with their care.

## Methods

### Study subjects and the organization of primary care

The study was carried out in two medium-sized municipalities located in southern Finland. The follow-up of diabetic patients in primary care had been organised in different ways in these municipalities since the early 1990’s. In the town of Kouvola (population 31,399), the primary diabetes care was based on general practitioners (Figure 1). In Nurmijärvi (population 35,922), the care of T1D patients and many of the T2D patients with enhanced insulin treatment had been centralised to one or two primary care doctors with several years’ clinical experience in diabetes care. Most T2D patients were, however, followed up by their own GPs with the possibility of consulting the diabetes-oriented GP. In the centralised system of Nurmijärvi all diabetic patients with increased risk of feet problems could see a foot therapist regularly with public funding or, in acute situations, when needed. In Kouvola there was no such facility. In Nurmijärvi more working time of the diabetes nurses was allocated to diabetes care (56 hours/week in Kouvola, 74 hours/week in Nurmijärvi). Neither municipality had a computerised recall system for reminding patients of the next control visit. In Nurmijärvi the diabetes team goes through the list of insulin treated diabetic patients manually 1–2 times yearly and takes contact by phone with those who are suspected to be dropouts and are deemed to have a big risk of complications. Kouvola bought retinal photographing from a private company, which has a well-working recall system. Nurmijärvi performed retinal screening with its own camera. The Nurmijärvi model was less expensive but there had been problems in setting up an automatic recall system.

In Nurmijärvi, most insulin pump therapies were initiated and followed up in PHC, whereas in Kouvola the pumps formed part of specialist-level T1D care. Insulin pump therapy is still quite rare in Finland and most T1D patients use multiple daily injections (MDI). In Nurmijärvi, seven (4%) of the 170 T1D patients were on pump therapy in the beginning of year 2005 and 33 (19%) at the end of year 2010. Insulin pump therapy is at least twice the price of MDI in the beginning – the eventual savings come with a delay of years or decades as a lower risk of diabetic complications [7-9]. Still today (November 2013) insulin pumps are a rarity in diabetes care in Kouvola (4% of T1D patients).

The diabetic populations of the two municipalities resemble each other: there are no significant differences between the mean ages of the patients in the different diabetes groups (44.1 years in Kouvola and 44.4 years in Nurmijärvi in T1D and 66.1 years in Kouvola and 64.4 years in Nurmijärvi in T2D at the beginning of the study) and the number of diabetic patients in the municipalities is amazingly similar. In both municipalities, 64.1% of the population was in working age (aged 16 to 64) in 2005, but in Nurmijärvi there were somewhat more children (about 25 vs. 15%) and fewer elderly people (about 10 vs. 20%) [10]. There were, however, no statistically significant differences in the patients’ ages at the time of diagnosis or in the durations of the disease between the target populations. More detailed information about the patient data collection and the background data of the target populations has been published elsewhere [11]. During the six years under review the total costs of public PHC (per inhabitant; including dental care) were 4–35% higher and the total costs of public specialist care 13–21% higher in Kouvola than in Nurmijärvi.

**Figure 2:**
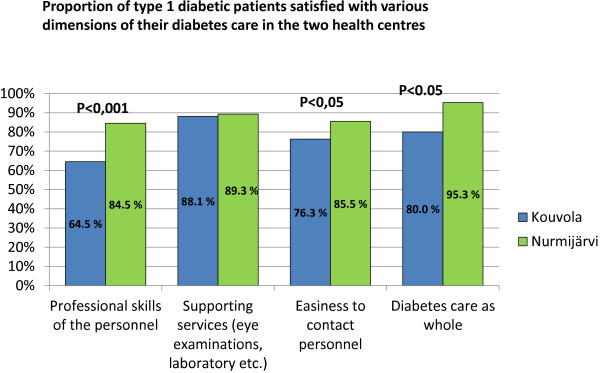
Proportion of type 1 diabetic patients satisfied with various dimensios of their diabetes care in two health centres.

The target population of this analysis is shown in Figure 1. The data on hospital care and specialist consultations due to diabetes and diabetes related diseases during the six years was collected from the National Discharge Register (HILMO) maintained by the Finnish National Institute for Health and Welfare. The HILMO register includes individual-level data on inpatient care in PHC and private health care as well as on all types of specialist care (outpatient and inpatient) on secondary and tertiary care levels. In regard of the outpatient care in PHC, the period of review was one year (2005) because this data could not be analyzed automatically. These results were multiplied by six and corrected to the price level of 2010 in the final analysis.

Altogether 1632 (69.0%) of the recruited patients signed an informed consent to examine their patient records, and their clinical and biometric data was collected and used in the final analysis. However, the extent of the use of health services by all the 2365 diabetic patients could be calculated on the basis of register linkages.

The diabetes type (type 1 or 2) was classified based on the data on the patients’ records in the hospital, the primary health care centre or other health care unit responsible for the follow-up. The number of diabetic patients was very similar in the two municipalities (171 T1D patients in Kouvola and 170 in Nurmijärvi; 958 T2D patients in Kouvola and 932 in Nurmijärvi). Forty one patients (1.7% of all) diagnosed with secondary diabetes (caused by pancreatitis or resection/trauma of the pancreas) with varying residual insulin excretion capacity were excluded from the final analysis (Figure 1). There were still 93 patients whose type of diabetes remained totally unknown because of lacking data in registers and no consent for examining their patient records. They were similarly excluded from the final analysis. This fact had, however, only minimal influence on the results, because these patients had not needed specialist level care during the study years. Half of the excluded patients (66) were from Kouvola and half (68) from Nurmijärvi.

### Use of specialist consultations and hospital care

In this evaluation, only visits or treatment periods due to diabetes or diabetes-related diseases were included in the analysis. All episodes related to psychiatry, gynecology, oncology etc. with no clear connection to diabetes were excluded. Typical included diagnoses were e.g. diabetic retinopathy, nephropathy, coronary heart disease, stroke and diabetic foot problems. The ICD-10 diagnoses of the PHC outpatient visits were not recorded in the HILMO register during the period under review. Every visit was analyzed afterwards by the study nurses and included in the study if the main reason for the visit was the patient’s diabetes or its complications.

### Quality of follow-up

We analyzed how well the current Finnish guidelines for the follow-up of diabetes were observed [6]. The time of the latest foot and retinal examinations, the blood pressure recording and the BMI (the body mass index) measurements and the analysis of overnight albuminuria, HbA_1c_ and LDL-cholesterol were determined and the results were compared between the two municipalities. There are many reasons why the existing guidelines for care are not always totally followed [12,13]. The reasons may depend on the patient, the doctor or the organisation.

### Patient satisfaction

50 randomly selected type 1 diabetic patients from both municipalities were asked to answer a 13-item questionnaire, where they evaluated their satisfaction to various parts of their diabetes care in their health care centres. The questions concerned the laboratory services, eye examinations, foot care, nutritional therapy, professional skills and availability of the diabetes nurses and doctors etc. All the questions had four options to be chosen from:

1) very satisfied

2) rather satisfied

3) rather unsatisfied

4) very unsatisfied

We excluded the totally neutral alternative like ‘no opinion’ in order to get stronger opinions. The patients returned their questionnaires anonymously in envelopes with only code numbers by which secretaries, who were not involved in the study, could send a reminder to those who had not answered the first posting. These secretaries also opened the envelopes and calculated the results. Altogether we got 41 answers from Kouvola and 43 from Nurmijärvi. For the analysis, we combined the questions in four categories: the skills of the personnel, the supporting services, the easiness to contact the personnel and the diabetes care as whole. We also combined two first alternatives as ‘satisfied’ and the two latest alternatives as ‘unsatisfied’ (Figure 2).

### Costs of diabetes care

The costs of care on the specialist level were calculated by means of DRG (diagnosis-related grouping) -based prices, which are used in the invoicing of the municipalities in Helsinki University Hospital (HUS) district and in the national statistics for costs of specialised health care services. The average DRG prices in university hospitals were ten per cent higher in comparison with the central hospital and private hospital level. The DRG prices in local hospitals were five per cent lower in comparison with the price level in central hospitals. The nearest and most frequently used hospital in Kouvola was classified as a local hospital while in Nurmijärvi it was classified as a central hospital with higher DRG prices. In the most serious cases, Kouvola’s patients were treated in the central hospital of Kotka whereas in Nurmijärvi they were referred to Helsinki University Central Hospital.

The costs in primary health care were based on the Finnish APR patient classification for primary care outpatients (Ambulatory and Primary Care Related Patient Groups), a grouping system equivalent to DRG [14]. It has been used during the last ten years in several Finnish municipalities covering the health services of over one million inhabitants. The APR has been routinely used in Kouvola Health Centre for managerial reporting of the produced services and costs for the past four years. The present study used the common standard unit prices of outpatient visits and hospital care days in PHC for both municipalities (€228.85/hospital day, €88.95/doctor visit and €46.98/diabetes nurse visit). All costs were fixed at the 2010 price level.

### Statistics

The differences between the costs of diabetes care as well as the mean levels of the laboratory parameters and blood pressure were compared using the Mann–Whitney test for two independent non-parametric samples, because the outcomes did not distribute normally. The other comparisons between the patients in the two municipalities were done with Chi-Square testing. In the analysis of the patient satisfaction questionnaire we also used the Bonferroni correction.

### Ethics

The Ethics Committee of the Department Internal Medicine in Helsinki Uusimaa Hospital District approved the study protocol and the Helsinki-Uusimaa and Kymenlaakso Hospital Districts granted permission to collect clinical data from patient records and ambulance registers. Moreover, all of the patients were asked for their consent to the use of their patient records. The HILMO register was used with the permission of the Finnish National Institute for Health and Welfare.

## Results

### Use of specialist consultations, inpatient and outpatient care

Both T1D (133, 77.8%) and T2D (528, 55.1%) patients in Kouvola had more outpatient hospital visits during the years 2005 – 2010 compared with patients in Nurmijärvi (101, 59.4%, p < 0.001 and 340, 32.9%, p < 0.01, respectively). There was no marked difference in the proportion of patients with T1D as regards the use of hospital beds (37.4% in Kouvola and 32.9% in Nurmijärvi) or in the average number of hospital admissions of T1D and T2D patients.

### Quality of diabetes care in PHC

A larger number of T1D patients living in Nurmijärvi (105, 61.7%) were followed up in their health centre (p < 0.05) than in Kouvola (84, 49.1%). As regards these patients, the Nurmijärvi patients’ follow-up included significantly more frequently the recommended recordings of the BMI, measurements of LDL-cholesterol and overnight urinary albumin excretion rates and clinical feet examinations than the follow-up of patients in Kouvola (Table 1).

**Table 1:** The coverage and the quality of the diabetes care in the two municipalities

**Examination/measurements**	**Type 1 diabetes**				**Type 2 diabetes**			
	**Examined (%)**		**Values (Means ± SD)**		**Examined (%)**		**Values (Means ± SD)**	
	**Kouvola**	**Nurmijarvi**	**Kouvola**	**Nurmijarvi**	**Kouvola**	**Nurmijarvi**	**Kouvola**	**Nurmijarvi**
LDL cholesterol (mol/l)	71.4	84.8	2.60 ± 0.56	2.57 ± 0.84	70.0	78.5	2.69 ± 0.79	2.56 ± 0.74
(During the latest 1.5 years)	(p < 0.05)		(n.s.)		(p < 0.01)		(n.s)	
BMI	51.2	77.1	24.7 ± 3.5	26.3 ± 5.0	69.5	61.3	30.4 ± 5.4	32.5 ± 6.1
(During the latest year)	(p < 0.01)		p < 0.05		(p < 0.01)		(n.s.)	
HbA1c (%)	89.3	94.3	8.16 ± 1.28	8.20 ± 1.28	93.2	90.7	7.09 ± 1.11	7.19 ± 1.11
(During the latest year)	(n.s.)		(n.s.)		(n.s.)		(n.s.)	
RR (mmHg)	83.3	90.4	140 ± 19/80 ± 10	140 ± 18/83 ± 10	91.2	87.0	144 ± 18/80 ± 10	146 ± 17/83 ± 10
(During the latest year)	(n.s.)		(n.s.)		(p < 0.05)		(p < 0.05/syst., p < 0.01/diast.)	
nU-alb	58.3	74.3			35.2	37.1		
(During the latest 1.5 years)	(p < 0.01)				(n.s.)			
Retinal photographing	73.8	53.3			49.2	35.1		
(According to national recommendations)	(p < 0.05)				(p < 0.01)			
Examination of the feet	44.0	88.0			43.5	43.2		
(During the latest 1.5 years)	(p < 0.01)				(n.s.)			

Only the patients who announced themselves to be in the follow-up of the health centres were included in the evaluation of the proportion of examined patients.

The T2D patients were mostly followed-up in their health centres in both municipalities (60.2% in Kouvola and 66.0% in Nurmijärvi). LDL-cholesterol was more often measured according to the guidelines in Nurmijärvi than in Kouvola (p < 0.01), whereas blood pressure measurements (p < 0.05) and retinal photographs (p < 0.01) were taken more systematically in Kouvola as shown in Table 1.

There were no statistically significant differences in the results of the essential laboratory tests between the study municipalities (Table 1). It is, however, worth mentioning that in both municipalities the average HbA_1c_ -level in TID was lower than the national Finnish level, which has been about 8,5% (69 mmol/mol) from decade to decade [2,3].

In Nurmijärvi the mean HbA_1c_ of T1D patients was 8.20% and in Kouvola 8.16%. The means of LDL cholesterol in T1D were 2.57 mmol/l in Nurmijärvi and 2.60 in Kouvola. Respectively, the mean values for LDL in T2D were 2.56 mmol/l in Nurmijärvi and 2.69 mmol/l in Kouvola. The means of blood pressure values of T1D patients measured during the first year of the study were 140/83 in Nurmijärvi and 140/80 in Kouvola (n.s.). In T2D the results were 146/83 in Nurmijärvi and 144/80 in Kouvola (p < 0.05).

### Patient satisfaction

The T1D patients of Nurmijärvi were more satisfied with the diabetes care in their health centre than T1D patients living in Kouvola (Figure 2), but both patient groups were similarly satisfied with the professional skills of the diabetes nurses.

### Costs of diabetes care

**Figure 3:**
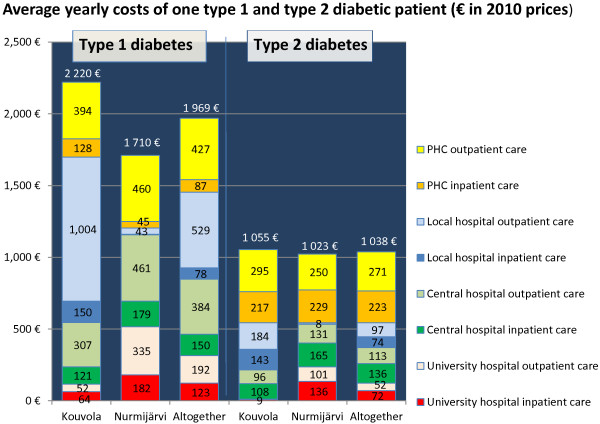
Average yearly costs of one type 1 and type 2 diabetic patient (€ in 2010 prices).

The costs of T1D were estimated with two methods. In the first analysis the costs of all specialist health care of T1D patients were €1,974/pt/year in Kouvola and €1,742/pt/year in Nurmijärvi. In the second analysis we excluded the costs of the users of the most expensive specialist care, including patients with diabetic nephropathy and chronic dialysis treatment (over €100,000 during the six years examined) (Figure 3). After the exclusion of coincidental variation caused by the very expensive patients, the yearly costs per T1D patient were €1,698 in Kouvola and €1,205 in Nurmijärvi. There was no difference in the costs of the diabetes care of T1D patients in PHC between the two municipalities. Altogether the total annual costs of one T1D patient were €510 lower in Nurmijärvi than in Kouvola. The difference in annual costs would have been €604 if the prices of specialist outpatient and inpatient care had been calculated at the central hospital level in both municipalities.

The costs of T2D patients were similar in both municipalities (Figure 3). The total annual cost was about €1000/pt, and there was only a slight difference of €32/pt/year for the benefit of Nurmijärvi. With similar specialist care prices the difference would have been €71/pt/year.

## Discussion

Our study indicates that it is sensible to organise the diabetes primary health care of T1D patients. If the T1D patients were followed up by GPs with good experience and interest in diabetes care, fewer special care consultations would be needed and a larger proportion of patients would get diabetes care from the local health centre. T1D patients themselves also seem to be more satisfied with the diabetes care provided by their local health centre. Centralised diabetes care in PHC also resulted in cost savings. The national guidelines for the treatment of T1D were better followed in centralised diabetes care, but the quality of diabetes care was similar in the two differently organised primary care models as regards glycemic control, blood pressure values and LDL-concentration. One problem for this study was that the diagnoses for outpatient visits in PHC were not yet entered into the electronic patient records. The quality of visits had to be manually assessed, which caused a lot of routine work.

Our study also revealed that the more centralised care of T2D in PHC resulted in less need for specialist consultations. However, there were no consistent differences in the quality of diabetes care in T2D patients whether followed up by regular GPs or to some extent by GPs with more experience in diabetes care. Moreover, the overall costs of diabetes care of T2D patients were almost similar in the different diabetes care models. The incidence of T2D is so high that every PHC doctor is more or less experienced in its care, and the difference in experienced and less experienced PHC doctors’ skills has less effect on the outcome of T2D patient care.

It is very challenging to compare the effects of differently organised diabetes care models in PHC. The proportion of the diabetic patients (even T1D) followed up within occupational or private health care varies in the municipalities. Furthermore, the use of different laboratory methods in the assessment of HbA_1c_ may obscure differences in the quality of diabetes care between the study cohorts [15]. However, the means and medians of the HbA_1c_-values of the T1D patients in both municipalities were lower than the Finnish values on average [2,3]. The Finnish results of T1D care measured with HbA_1c_ are not very good but still comparative with other countries [16]. Benchmarking is very problematic before we have national diabetes registers in all countries and before laboratory results are truly comparative and automatically available on the whole population from data systems [17,18].

The strength of our study is that the diabetes care under review had already been organised in the same way for 15 years in the two municipalities before our study began and that the data on the use of specialist care of diabetes was collected from a period of six years to minimise occasional changes in the use of specialist consultations. The patient cohorts in both municipalities were identified from the customer lists of the public cost-free distribution points of diabetes supplies. In Finland practically every diabetic patient uses the possibility for cost-free diabetes supplies. Therefore the original study population represented the whole adult diabetic populations of the municipalities. In both municipalities, a similar proportion of all diabetic patients participated in our study, and the sizes and demographics of the final study populations were comparable.

The primary target for us was to compare the use and costs of specialist consultations and hospital care due to diabetes and its complications in two municipalities with different models of PHC organisation. The diagnosis-related database of our study includes all inpatient care in PHC and private health care as well as outpatient and inpatient specialist care given in local, central and university hospitals. Kangas et al. have previously calculated the overall costs of diabetes care in Finland [19]. According to his data, diabetes with its complications is by far the most expensive disease for the Finnish health care system. However, our study is the first follow-up study to estimate both the use and the costs of health services in PHC and specialist health care.

Based on our calculations, Nurmijärvi saved annually around €86,700 with its centralised care of T1D patients as compared to the decentralised care of T1D patients, in which a larger proportion of patients are followed up by specialist care units. These savings did not impair the quality or T1D patients’ satisfaction with their care. The cost savings calculation in Nurmijärvi was based on only 170 T1D patients. It is well known that the number of T1D patients is steadily increasing in Finland [1]. If the Nurmijärvi model (the centralised model) were applied more widely in the care of T1D patients in Finland, the annual savings would be over 4 million euros per 10,000 T1D patients. These figures indicate that it could be not only useful but also cost-effective to assign the care of T1D to GPs in PHC who have good interest and experience in diabetes care. The system is also applicable to the care of some other chronic diseases.

The findings can also be expressed in another way. In a municipality of a population of around 40,000, the centralised primary health care of T1D and complicated T2D patients would yield the yearly savings equal to the annual salary of one GP.

The lower number of specialist care consultations in Nurmijärvi can probably be attributed to consultations between general GPs and diabetes-oriented GPs. Overall, T1D patients in both municipalities were very satisfied with the diabetes care provided. The PHC personnel’s professional skills were rated higher in the centralised system. Since there were no differences in the professional skills of the diabetic nurses, the differences in the skills of GPs may have played a major part in patient satisfaction. Especially young T1D patients may find it easier to contact the familiar personnel at the nearby diabetes service with their minor daily problems.

However, being dependent on a little group of experienced employees, the centralised T1D care system is vulnerable. Diabetes nurses try to manage during the diabetes-oriented doctor’s holidays - with the support of other GPs, if needed - and a sudden absence for any reason would be a major problem. The training of a new employee inevitably takes months.

## Conclusions

Our data suggests that the diabetes care of T1D patients can be arranged in primary health care without compromising good quality and patient satisfaction. It can yield cost savings through less frequent need for specialist consultations if a GP working at the health centre has good experience in and motivation for diabetes care. In regard of T2D patients, the model of providing diabetes care in PHC had a similar effect on the quality of care and reduced the yearly costs by 3%. These findings should encourage primary health care units to critically evaluate their present model of diabetes care.

## Abbreviations

PHC: Primary health care; HC: Health care; T1D: Type 1 diabetes; T2D: Type 2 diabetes; BMI: Body mass index; GP: General practitioner; DRG: Diagnosis related grouping; APR: Ambulatory and primary care related patient groups; MDI: Multiple daily injections.

## Competing interests

The authors declare that they have no financial competing interests. The first author has a long working history in the centralised diabetes care system of Nurmijärvi.

## Authors’ contributions

This study is the main part of MH’s doctoral thesis. He has written the manuscript. ML is a health economics researcher and has collected the data from the HILMO register. TS is one of the two supervisors of MH’s doctoral work and, as an endocrinologist, has frequently critically reviewed the study and its results. AH has organised and taken part in the analysis of the data. OE is the other supervisor of MH’s doctoral work and has had a major role in the planning of the study. OE is specialised in the management and economics of health care organisations. All authors have read and approved the final manuscript.

## Authors’ information

MH works as a GP in Nurmijärvi Health Centre and as a researcher in the doctoral school called the Network of Academic Health Centres, University of Helsinki (AcaHC). The objective of AcaHC is to promote scientific work and the development of operational practices based on science as a part of the work in primary health care and to enhance the attractiveness of primary health care among experts who have received scientific education. The research ideas arise from the daily work in health centres.

OE has also specialised in general practice and public health and works as the coordinator of AcaHC.

TS has a long career as an endocrinologist and diabetologist in Helsinki University Hospital.

ML is a Doctor of Science (Technology) and a health economics researcher in Aalto University.

AH is a Master of Science (Economics) and works as a data analyst in Qvantel Oy.

The aim of our present study is to find a cost-effective way to organise the care of diabetes in public health care as costs keep growing.

## Pre-publication history

The pre-publication history for this paper can be accessed here:

http://www.biomedcentral.com/1472-6963/14/26/prepub

## References

[B1] Karvonen M Viik-Kajander M Moltchanova E Libman I LaPorte R Tuomilehto J Incidence of childhood type 1 diabetes worldwide Diabetes Care 2000 14 1516 1526 10.2337/diacare.23.10.1516 11023146

[B2] Valle T Koivisto V Reunanen A Kangas T Rissanen A Glycemic control in patients with diabetes in Finland Diabetes Care 1999 14 575 579 10.2337/diacare.22.4.575 10189534

[B3] Valle T The balance of care of diabetic patients in Finland in 2009 – 2010: the Finnish diabetes association 2010 DEHKO 5 http://www.diabetes.fi/files/1488/DEHKO-raportti_2010_5_Diabeetikkojen_hoitotasapaino_Suomessa_vuosina_2009-2010.pdf

[B4] OECD OECD economic surveys: Finland 2012; Ch2 2012 OECD Publishing http://dx.doi.org/10.1787/eco_surveys-fin-2012-en

[B5] Ho M Marger M Beart J Yip I Shekelle P Is the quality of diabetes care better in a diabetes clinic or in a general medicine clinic? Diabetes Care 1997 14 472 475 10.2337/diacare.20.4.472 9096962

[B6] Diabetes current care summary 16.11.2011 http://www.kaypahoito.fi/web/kh/suositukset/naytaartikkeli/tunnus/ccs00032

[B7] St Charles M Lynch P Graham C Minshall ME A cost-effectiveness analysis of continuous subcutaneous insulin injection versus multiple daily injections in type 1 diabetes patients: a third-party US payer perspective Value Health 2009 14 674 686 10.1111/j.1524-4733.2008.00478.x 19171006

[B8] Nørgaard K Sohlberg A Goodall G Cost-effectiveness of continuous subcutaneous insulin infusion therapy for type 1 diabetes Ugeskr Laeger 2010 14 2020 2025 20594535

[B9] St Charles ME Sadri H Minshall ME Tunis SL Health economic comparison between continuous subcutaneous insulin infusion and multiple daily injections of insulin for the treatment of adult type 1 diabetes in Canada Clin Ther 2009 14 657 667 10.1016/j.clinthera.2009.03.013 19393856

[B10] SOTKAnet statistics and indicator bank [http://uusi.sotkanet.fi/portal/page/portal/etusivu]

[B11] Honkasalo M Elonheimo O Sane T Severe hypoglycaemia in drug-treated diabetic patients needs attention: a population-based study Scand J Prim Health Care 2011 14 165 170 10.3109/02813432.2011.580090 21675825 PMC3347964

[B12] Ijäs J Alanen S Kaila M Ketola E Nyberg S Välimäki M Mäkelä M Primary care guidelines: senior executives’ views on changing health centre practices in hypertension treatment Scand J Prim Health Care 2009 14 202 207 10.3109/02813430903438726 19929184 PMC3413911

[B13] Juul L Sandbaek A Foldspang A Frydenberg M Borch-Johnsen K Lauritzen T Adherence to guidelines in people with screen-detected type 2 diabetes, ADDITION, Denmark Scand J Prim Health Care 2009 14 223 231 10.3109/02813430903279117 19929182 PMC3413914

[B14] Elonheimo O Myllymäki K Linna M Eroja Kouvolan omien ja ulkoistettujen terveysasemien palveluntuotannossa Suom Laakaril 2011 14 1103 1112 English Summary

[B15] Honkasalo M Elonheimo O Sane T HbA_1c_-määritysten tuloksissa on tasoeroa [Differences exist between the levels of HbA_1c_ determinations] Suom Laakaril 2007 14 1609 1612 English Summary

[B16] McKnight JA An international collaboration to compare glycaemic control among people with type 1 diabetes http://www.abstractsonline.com/plan/ViewAbstract.aspx?mID=3290&sKey=c9f7e022-911a-4893-8a74-13f557d34568&cKey=e5059503-5979-41fe-9233-e8e86eb9b7d8&mKey=7e87e03a-5554-4497-b245-98adf263043c, OP07, EASD49 Barcelona, 2013

[B17] O’Mullane M McHugh S Bradley CP Informing the development of a national diabetes register in Ireland: a literature review of the impact of patient registration on diabetes care Inform Prim Care 2010 14 157 168 21396238 10.14236/jhi.v18i3.768

[B18] Maizlish NA Shaw B Hendry K Glycemic control in diabetic patients served by community health centers Am J Med Qual 2004 14 172 179 10.1177/106286060401900406 15368782

[B19] Kangas T Aro S Koivisto VT Salinto M Laakso M Reunanen A Structure and costs of health care of diabetic patients in Finland Diabetes Care 1996 14 494 497 10.2337/diacare.19.5.494 8732715

